# Distributed Service-Based Approach for Sensor Data Fusion in IoT Environments

**DOI:** 10.3390/s141019200

**Published:** 2014-10-15

**Authors:** Sandra Rodríguez-Valenzuela, Juan A. Holgado-Terriza, José M. Gutiérrez-Guerrero, Jesús L. Muros-Cobos

**Affiliations:** Software Engineering Department, University of Granada, C/Periodista Daniel Saucedo Aranda s/n, Granada 18071, Spain; E-Mails: sandra@ugr.es (S.R.-V.); jmgutierrez@ugr.es (J.M.G.-G.); jesusmuros@ugr.es (J.L.M.-C.)

**Keywords:** SOA, service composition, data fusion, Internet of Things, pervasive computing

## Abstract

The Internet of Things (IoT) enables the communication among smart objects promoting the pervasive presence around us of a variety of things or objects that are able to interact and cooperate jointly to reach common goals. IoT objects can obtain data from their context, such as the home, office, industry or body. These data can be combined to obtain new and more complex information applying data fusion processes. However, to apply data fusion algorithms in IoT environments, the full system must deal with distributed nodes, decentralized communication and support scalability and nodes dynamicity, among others restrictions. In this paper, a novel method to manage data acquisition and fusion based on a distributed service composition model is presented, improving the data treatment in IoT pervasive environments.

## 1. Introduction

The main idea of the Internet of Things (IoT) is the pervasive presence around us of many smart things or objects. These objects can be real-world physical devices as sensors, actuators or devices, as well as data resources that are able to interact and cooperate with their neighbors to reach common goals [1].

IoT has direct applications and involves new paradigms of software development in fields, such as home-automation, ambient assisted living, healthcare, smart-cities, industrial management and artificial intelligent computing [2]. IoT promotes that all objects of the real world, at home, at the office and everywhere, are interconnected and provide new applications and functionalities with an autonomous, smart collaborative behavior. However, the development of IoT software infrastructures has new problems to overcome related to networking and composition aspects. The classical distributed software model does not work well with a network of heterogeneous devices that are capable of collaborating among them to provide higher value-added functionality to end users. Thousands, even millions, of devices should be identified, having a well-defined functionality and being connected to a network, as well. IoT objects are resource-constrained devices in terms of both computation and energy capacity. Then, the proposed solutions must be lightweight applications that use efficiently the resources and manage the scalability and interoperability of devices [3].

The big heterogeneity of hardware devices used in IoT makes it suitable to adopt a middleware layer, which guarantees scalability and interoperability. Service-oriented architecture (SOA) is becoming the most widespread approach to implement middleware for distributed systems [4]. However, the application of SOA principles without an appropriate setting of non-functional requirements in terms of quality attributes (e.g., performance or bandwidth) and system configuration (e.g., involved technology or platforms) provides useless systems that can hardly guarantee the robustness, reliability, availability and scalability properties required in current pervasive spaces. The idea of assembling application components into a network of loosely coupled services to create flexible and dynamic processes with agile applications running on different computing platforms reinforces the role of the service as the main abstraction to support the development of distributed applications.

The success of IoT application development will be strongly linked with the cooperation and collaboration among heterogeneous networked embedded devices through services. Real-world devices in the next generation Internet will be able to share their functionality and cooperate with other components dynamically. An example of a real IoT scenario could be a recommender system (RS), which predicts items or ratings of items that users are interested in. The RS will combine and fuse the information provided by sensors as heterogeneous data to obtain new information and to suggest something interesting to final users [5].

Data fusion processes have been traditionally carried out in a centralized fashion using powerful servers that analyze, apply reasoning and perform inference of new knowledge with data [6]. In this paper, we propose a new method to manage data acquisition and fusion based on a distributed service composition model applied to an IoT case study. It provides a high level model of representation, which abstracts the underlying complexity and heterogeneity of devices that it is typical in IoT scenarios. Developers can model the IoT scenario using services and the interaction between services using a well-defined service composition model. The service composition model allows us to design an IoT system from basic elements, devices, to high level software units, services with complex functionality, in a distributed fashion. The service interaction modeled by using the service composition model adds relevant QoS properties to the system, such as bounded execution time or latency. If developers know bounded values of execution time in the request of service operations, they can add soft-real time restrictions to the system execution. Considering these soft-real time properties, developers can analyze their systems before to deploy them into real scenarios and optimize the use of the whole system defining, for instance, the most appropriate sampling time of specific services.

The proposed idea has already been discussed in other contexts related to IoT before, such as environmental monitoring and energy management [7], logistics [8] or healthcare [9]. All of the applications proposed in these studies set a unified identification and communication framework between smart sensors and the transmission of data via the Internet. Data transmitted from sensors are then processed and managed by a server or a specific powerful computing node in a cloud. However, they hardly ever argue about the possibility to compute the information at different levels on devices, even in sensors themselves. The solution presented in this paper claims that data fusion in IoT has to be processed distributively following a service composition model in every device according to its computing capacity over the IoT network, without needing a centralized server.

The applicability of the proposed method and the consequences in the execution of the system are shown in detail with a case study focused on weather forecaster. However, there are other situations of real life where the proposed solution can be applied. For example, in a home automation scenario, the information from sensors can be combined with user preferences to accommodate the house state, such as temperature, illumination, *etc.* In an industrial scenario, where the production can be affected by several situations, the proposed method can be applied, as well. During the procedure, sensors may obtain and process information, whereas high level services may analyze the data. If a critical situation is detected, other services will be started to inform the responsible person that something has happened.

The method proposed has several relevant properties desirables in IoT environments. It is scalable, since new services can be easily added to the system and then collaborate with the rest of services, aiming at a common goal. The scalability of the system is based in the underlying SOA middleware, which support the service composition model. It is also decentralized, *i.e.*, each service has available all the information about its required services in order to process that information according to its computing capabilities, and is efficient, because the correctness of the collaboration between services is ensured by construction. Hence, the system can be analyzed without any complex verification procedure and to detect the possible errors regarding the model. Moreover, it favors a lightweight composition procedure, because services do not need to know which service is requesting its functionality, only what the required services are.

The rest of the paper is organized as follows. Section 2 introduces the research background related to this work. Section 3 details the main aspects of the data fusion method using the service composition model presented in this paper. Section 4 details a case study, analyzing the hardware devices used, the services implemented and the results obtained after running the test. Finally, Section 5 shows the future research lines and the conclusions of the work.

## 2. Background

Pervasive computing and IoT are changing the way we live. There is a virtual world of services with which we interact at work, at home and even in our relationship with other people. The application of IoT goes from ambient assisted living, industrial equipment to the healthcare domain [10], including sensing, data collection and monitoring personal, health and environmental parameters. Fused and combined information from sensors embedded in mobile devices can be used by IoT applications to determine the user's situation and to build adaptive context-aware services [11].

Considering data and functions as services is well suited in pervasive computing and IoT [12]. The building of new collaborative services with complex functionality, data fusion algorithms applied to obtain context-awareness, the semantic representation of the information and the final applications can be seen as composite operations distributed between services over a well-formed middleware [13]. In this context, we analyze data acquisition and fusion methods using lightweight collaborative services. One of our research objectives is to give distributed support to, for instance, the fusion process, avoiding the centralized figure of the fuser using quality of service (QoS) properties to select the best service to carry out the functionality of an application.

SOA-based architecture implemented in mOSGi is used by Bernardos *et al.*, to develop context-aware applications over a framework called CASanDRA (Context Acquisition Services and Reasoning Algorithm) [14]. In the scope of the interconnected embedded devices, the Open Services Gateway initiative (OSGi) alliance has created several specifications to make easier the development of distributed applications. This specification defines a system that allows designing compatible platforms to share services [15]. All services can be executed and dynamically linked into a particular node or remotely among independent network nodes from OSGi 4.2. Other interesting approach is to use the Resource-Oriented and Ontology-Driven Development (ROOD) methodology based on the Model Driven Architecture (MDA) to carry out the development of Internet-oriented platforms [16]. In this case, the middleware is designed to manage and control directly network resources (instead of services) that are available and accessible through specific nodes in the network.

Wireless sensor network for healthcare [17] and, more specifically, body sensor networks (BSNs) are also very popular challenges related to IoT [18]. BSNs come with the promise to improve the quality of life and healthcare of disabled and elderly people and also to improve our daily routines, such as playing sports [19]. However, the distributed and changeable character of this kind of network has new challenges to solve. Research in the area of BSNs must cover low-level hardware design to higher level communication and data fusion algorithms, up to top-level applications [20]. Therefore, it is advisable to use a distributed service middleware to support scalability and reusability. Moreover, services and SOA-oriented middleware also are presented in IoT research projects that use semantic and ontologies to enrich the information with which the applications work [21,22].

QoS properties are also relevant in pervasive service platforms, and there are several research works that consider QoS properties in the composition process of their middleware platforms: Estévez-Ayres *et al.* define a hybrid approach for selecting services using real-time composition algorithms [23]; Chang and Lee consider a multi-criteria quality model in three dimensions, services, contents and devices, to define its composition methodology [24]; Moser *et al.* define non-deterministic QoS attributes to facilitate the domain-specific service selection [25].

In terms of communication, an effort to bring lightweight Internet connectivity to smart IoT objects is being carried out by several organizations, such as IETF (Internet Engineering Task Force) and the IPSO (Internet Protocol for Smart Object) Alliance [26,27]. In this sense, it has been designed several protocols to be used by constrained nodes in a lightweight fashion; such as CoAP (Constrained Application Protocol) [28], MQTT (Message Queue Telemetry Transport) [29] and CoSIP (Constrained Session Initiation Protocol) [30]. These protocols can be adopted in IoT environments.

A complementary approach to SOA-based systems has been also applied to multisensor data fusion based on multi-agent systems (MAS) [31]. In MAS, agents represent software entities that can be equivalent to services. In fact, since agents are autonomous, interactive and adaptive, a hierarchy of communicating agents can be conformed from basic agents till more abstract agents to accomplish a specific goal [32] in a similar way as a complex service in an SOA context encapsulates its functionality by the composition of simpler services. However, although SOA and MAS may share some particular objectives, their main focus seems to be different. Unlike services, agents are powerful in their communication capacity and interaction with other agents, allowing them to react to changes in the environment and adapting its behavior [33,34]. Then, the agent paradigm is best suited to give support to service-oriented systems [35,36]. The service-oriented system are focused on the structural part of the system profiting from the enhanced capacity to define syntactically systems, even from diverse organizations, while agents as active elements of the system are responsible for making smart, efficient and optimum use of defined services; *i.e.*, agents can determine the best way to compose services dynamically [37]. In our case, the proposal in this paper is more concentrated in determining how the system can be structured into service entities to be integrated correctly by the service composition model. Moreover, it analyzes how this service composition model can be the support for the development of data fusion processes in a distributed fashion considering the restrictions imposed by the QoS properties of each service, especially in IoT environments. In a scenario where a service consumer (or even an agent) can consume the functionality or resources provided by services statically or dynamically, the proposed model addresses several requirements that help to ensure the correction of the whole system (e.g., scalability).

## 3. Data Fusion Using Service Composition Model

Next generation Internet comes with the promise to integrate several technologies and communication systems with the aim of building an intelligent abstraction of the information around the world. On the other hand, machine-to-machine (M2M) communication systems combine multiple sensor interactions and merge the information obtained by sensors applying several data fusion algorithms [38].

In this paper, the distributed data fusion mechanism is based on a well-defined service composition model using device services to encapsulate the functionality and restrictions of physical devices. Device services are not only abstract physical devices, but they also provide a high level model of identification, characterization and communication among services. These aspects are very relevant in IoT scenarios, where the amount of devices can be huge. Furthermore, in contrast to hardware-based approaches, a change in the functionality of a service should not affect physical devices, because the deployment is controlled at a high level of abstraction, without repercussions at the physical level.

To carry out data fusion, we have to distinguish mechanisms used for acquiring data to be fused with respect to specific algorithms applied to produce new data. In this paper, we have worked with the first one, *i.e.*, mechanisms for data provision. In general, the papers in the bibliography are more focused on the determination of algorithms for data fusion, investigations about its complexity and the provision of good estimators and classifiers [39].

Traditionally, data fusion is processed by a software/hardware component, which has direct access to external data sources, invoking API functions or reading digital data transmissions through system buses. These components have to run by imposing a sustained data provision and also satisfying specific restrictions (e.g., real-time communication). The implementation of a time-out facility helps to control both above issues and determines when fusion is not possible (e.g., data is not available). Therefore, this mechanism has to enable the use of time parameters to specify the time interval required by an operation to be executed, providing the possibility of designing deterministic algorithms with soft real-time requirements.

Instead of implementing in-house or legacy components, the application of a distributed technology for data fusion has some advantages with respect to traditional approaches. A distributed paradigm supplies a well-established communication model (e.g., message passing primitives) with the control of networks fails, giving a reliable binding mechanism with external data sources. In addition, the computing processing is shared between devices instead of performing all on a single device or server.

The SOA paradigm benefits the overall building of a system, since the system can be seen as a group of interconnected low coupled services. Each service is in charge of its part of functionality and must maintain its produced data. Besides, the service composition model gives a natural way to implement a distributed data fusion mechanism.

### 3.1. IoT Services

The service is the main element of the SOA paradigm [40]. In general a service is an autonomous, self-contained component capable of performing specific activities or functions independently, which accepts one or more requests and delivers one or more responses through a well-defined, standard interface. From an IoT point of view, services can represent hardware devices, software resources and any other thing or object that can be identified and located in specific places.

An IoT service needs a set of elements to characterize itself with respect to the rest of services in the network. Formally, we can represent an IoT service by the five-tuple that we can show in Definition 1, where *Id_i_* is the identification, *Ps_i_* its purpose, *Ip_i_* the provided interface, *Ir_i_* the required interface and, finally, *At_i_* the set of attributes.

(1) $${\text{IoT service}}_{i}=<Id_{i},Ps_{i},Ip_{i},Ir_{i},At_{i}>$$

First of all, an IoT service needs identification, *i.e.*, a unique name/ID that identifies the service unequivocally. Each IoT service can have many runnable instances with the same functionality, but executed at different locations. The identification must be unique for each instance of the IoT service and readable by any other IoT services, although the service is not in execution. In order to identify univocally each IoT service, the identification is based on the uniform resource name (URN), a type of URI (Uniform Resource Identifier) that is intended to be persistent resource identifiers, independent of location.

Second, the IoT service has to specify its purpose, *i.e.*, what its functionality is or what the responsibilities of this service are. Service functionality can be expressed in terms of functional requirements using natural language, but it is preferred to use a description on the basis of a set of operations that can be provided to the rest of IoT services. These operations are public and are accessible for any other IoT service that requests them.

An IoT service is able to assume different roles, depending on how the interaction is set between two services, accordingly a SOA service. As the service provider, it is responsible of supplying its specific functionality by the invocation of a set of operations to any other IoT service, which would act as a service consumer. An IoT service can perform two types of operations: simple or composite. A simple operation is a single transaction that the service can perform by itself, *i.e.*, the service has all of the necessary resources to carry it out and it does not require interaction with any other service. In contrast, a composite operation involves, in addition, the execution of a set of operations on at least one service provider.

Third, the IoT service needs to specify the interface that defines the interaction capacity that an IoT service may have with other services. The interface used by the service to act as consumer is different than the interface used to act as provider. Therefore, the interface can be provided or required. The provided interface exposes the signature of the functions or operations accessible to other external services as a service provider. With the role of the service provider, it is responsible for receiving requests from other consumer services and then manages the execution of invoked operations, giving a response to them when necessary. At the opposite, the required interface includes the signature of functions or operations that an IoT service may invoke in other services as a consumer service. Then, both interfaces, required and provided, could be seen as the set of operations that service must run on its own service or on required services. The ports are the communication channels available to and from the service. In the scope of the provided interface, each operation has an independent port. This establishes a control and synchronization mechanism with respect to executed operations, because the service may receive different requests associated with the same operation from different consumer services. Each port at the required interface includes one of the operations requested in other services.

In addition, each service has other properties that restrict its execution and its applicability. These properties define the attributes of the service and should also be known by consumer services before using it. In general, they provide additional information about the service content (data, algorithm, software or hardware resource) and the service execution, but its configuration can also partially modify the behavior of the IoT service. Some of the service attributes are defined specifically for each service instance, whereas other ones are defined for all instances of a specific service.

### 3.2. Device and Fuser Services

In the context of data fusion, we can set two additional roles for IoT services: device services and fuser services. Device services encapsulate hardware resources (e.g., devices, sensors and actuators) that interact with the environment and provide operations to manage them. These services always act as service providers. In contrast, fuser services use the information provided by device services as data sources and fuse this information in order to infer new or filtered information to other service consumers. They can also collaborate with other services or carry out actions over the environment proactively, as well. Then, these services are composable by nature and can act as service consumers or service providers.

The use of device services gives us a way to harmonize the access to hardware resources (e.g., sensors and actuators), hiding their particularities to outside. A device service is responsible for including mechanisms to control and manage these hardware resources, which are addressed by operations defined in the provided interface *Ip(S_i_)*. Device services do not have required interface *Ir(S_i_)*, because they do not interact with other services to carry out their operations.

The device service has to offer extended information about hardware resources and service execution, which defines both of the device service attributes. On the one hand, knowing device data of hardware resources might be essential for the system engineer to build a specific fuser from device services, e.g., properties such as device calibration, device diagnostics, data quality of device readings in sensors or actions in actuators, types of transducers, manufacturers-related information, or where the device is connected. Thus, not only device data is transferred, but also the metadata that describes the device data [41]. Then, provided interface *Ip(S_i_)* of device services will include operations to consult the extended information of hardware resources behind the service and operations to modify or delete these ones when the device provides configuration capability.

On the other hand, the implementation of the service restricts the service execution and overall capabilities of that service. This implementation is obviously linked with the performance and the dependability of the hardware device. Therefore, the implementation of a device service must include the specification of information related to its execution, even with real-time constraints, e.g., the time involved in reading/writing the device or the frequency of updating the device.

The information captured in a real environment by device services have to be processed and interpreted before carrying out a proactive behavior or giving a result to the user. This is the role of the fuser services, which can invoke the operations of device services to get the data that will be fused and enriched to generate additional, new information. This information can be completely new or filtered from data sources.

In this kind of service, the service composition may have a special importance. Fuser services can act as service consumers, requesting data or demanding actions on device services, as well as service providers, offering fused data to other fuser services. Thus, services can be scaled building a composition of interacted services. These interactions among services define the data fusion mechanism based on a service composition model, which is detailed in the next section.

### 3.3. Service Composition Model

The service composition model depicts an abstract model of interactions among low-coupled, reusable services with the purpose to build scalable applications from the integration of such services. The use of a service composition model can benefit data fusion systems, since it provides a flexible mechanism to arrange data sources and fuser nodes and to perform fusion with them as shared resources. In this paper, the proposed data fusion mechanism is based on a well-defined service composition model [42,43]. Next, the mathematical formulation of service composition is presented.

In the above section, we have seen that services offer their functionality by means of operations. A service that only has simple operations is called a basic service; e.g., a device service. In contrast, there are services that implement composite operations, which invoke other services, they are called composite services; e.g., a fuser service. Composite operations are the base of the collaboration model and are defined in the required interface *Ir(Si)* of the service definition (see Definition 1).

A full pre-designed set of these composite operations defines the service composition map. Its awareness *a priori* before holding up the service determines a static service composition model, since the collaborative process is clearly predefined. In contrast, in a dynamic service composition model, the service and the invoked operations are selected at runtime using semantic information, ontologies or inference [44]. Although there exists a controversy about what (and what not) dynamic composition is, there are many research works that confuse dynamic composition with the dynamic selection of services. When information and functionality related to all services and their operations are known, dynamic composition will dynamically decide which services are able to achieve the requested functionality. In contrast, in dynamic selection of services, the composite functionality is established *a priori*, but the specific instance of the service that will execute the operation is selected at runtime between all of the available instances of the service. Dynamic composition is very powerful, but it also increases the complexity of the behavior adaptation at runtime. In the presented service composition model, if there are two or more instances of the same service, the specific instance used in the composite operation will be selected at runtime using its QoS properties. However, this is not dynamic composition. We know *a priori* the kind of services that we should use, but we choose at runtime the specific instance of each service that better accomplish the requirements. In our opinion, this process may be called dynamic selection of services and not dynamic composition.

An example of a QoS property that we use in the dynamic selection of services is related to the execution time of the operations. Each operation, simple or composite, has an estimated execution time. The execution time of an operation is fixed by the worst-case execution time (WCET), which represents the maximum possible value for this execution time. Each operation has its own WCET, which is determined using the WCET of the requested operations in the case of the composite ones. Furthermore, in composite operations, a bound for the networks communication delays is necessary to be added. This execution time is used as a QoS property to select the best available instance of the service to execute that operation. When an operation is invoked, the requester knows its maximum execution time and, hence, the maximum time that it has to wait to receive a response. This mechanism ensures executing operations with soft real-time quality properties.

Service composition has been modeled using graph theory. The composition map of a service is formed by composite operations, and the relation between these operations, invoker or requested, can be seen as a composition graph. Then, each composite operation *op* of a service *S* can be seen as a directed graph, as we can see in Definition 2, where:

(2) $${\text{G}}_{{\text{op}}_{\text{s}}}=\left({\text{o}}_{{\text{op}}_{\text{s}}},\text{V}(\text{G}),\text{L}(\text{G}),\text{E}(\text{G})\right)$$

- *o_ops_* is the main vertex of the graph and corresponds to the origin service *S* of the composite operation *op_s_*.
- *V(G)* is the set of vertices of the graph where each vertex represents a required service on which an operation is invoked from the composite operation *op_s_*.
- *L(G)* is the set of labels where each label embodies a requested operation in a required service of *V(G)*.
- *E(G)* is the set of direct edges between the origin vertex *o_ops_* and a destination vertex in *V(G)* labeled with an element of *L(G)*. Accordingly, each element of this set is defined by Function 3.

(3) $${\text{edge}}_{i}(o_{op_{s}},op_{i},v_{j})$$

In Function 3, *o_ops_* is the origin service and *op_i_* is the requested operation in the required service *v_j_*, verifiying that *E*(*G*) ⊆ *o x L*(*G*)*x V*(*G*).

The composition graph of a given service may contain calls to several services operations in the same composite operation, but these operations are performed in a sequential fashion, not nested. The depth of the composition graph of a service is always of one level, since a service starting a composite operation does not know if one of the operations in its required services will be composite, too. In this case, we could have a chain of composite operations allowing one to call an operation from inside another one. However, to ensure the execution time in composite operations, it is not possible to have cyclic calls of operations, *i.e.*, operations of the owner service cannot be required by an operation from its required services along the execution of the composite operation. Because of this, we define the function *complexDeg(G_ops_)* as the degree of complexity of an operation, which establishes the depth of its graph. A simple operation has degree of complexity zero, *i.e.*, *complexDeg(op) = 0*. The degree of complexity of a composite operation *G_ops_* is specified by Definition 4.

(4) $$\text{complexDeg}(G_{op_{s}})=\text{max}(\text{complexDeg}(op_{i}))+1\;\forall op_{i}\in L(G)$$

**Figure 1.**
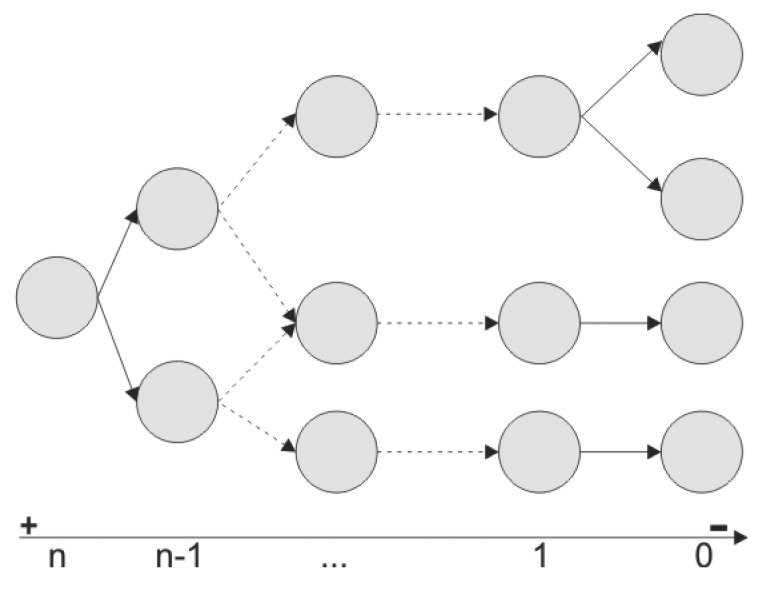
A graphical view reflecting how the degree of complexity of composite operation is increased.

We can say that the degree of complexity of a service is the maximum degree of complexity of all of the composite operations of its composition map plus one, as we can see in Figure 1. We have defined Axiom 5 based on the restriction imposed over the invocations between composite operations for the function *complexDeg(op)*. Let *op_v1_* be a composite operation in the service *v1* (origin vertex) and *op_v2_* a requested operation in service *v2* (destination vertex). Using this axiom, we can prove by transitivity the property of acyclicity of the service composition map.

(5) $$\text{edge}\left(o_{op_{v1}},op_{v2},v2\right)\to \text{complexDeg}(op_{v1})>\text{complexDeg}(op_{v2})$$

In a typical pervasive computing scenario, services can join and drop spontaneously at any time. It is important to have a robust composition model that ensures the correctness of the distributed communication. The complexity degree of services defined in the composition model presented allows us to determine the complexity of a single service or a full application that uses a lot of services. The degree of complexity of an operation gives us useful information about the collaboration capabilities of each service, and it also helps us to determine the time involved in the operation execution. Composite operations with a higher degree of complexity will have also higher WCET, because they must add to their own execution time the execution time of the requested operations.

The degree of complexity must be updated when composition map changes. This updating process is necessary to ensure the acyclicity of the composition graph. By means of the composition graph, we can design distributed data fusion algorithms based on collaborative services and delimiting their execution time as a QoS property.

### 3.4. Service-Based Middleware Platform

The service-based middleware used to implement data fusion in IoT is called Dynamic Open Home Automation (DOHA) [45]. The DOHA middleware provides support for the development of decentralized applications, which hide the communication complexities. DOHA enables developers to build applications based on a set of independent services, where new services can be added without knowing the implementation or operations of the rest of services already running on the underlying platform.

To test the applicability of the distributed data acquisition mechanism based on DOHA services, we have chosen an implementation of DOHA using DPWS (Device Profile Web Service) as the underlying platform. DPWS is a framework to develop lightweight web services, especially for embedded devices [46]. DPWS adds some restrictions to the standard specification of web services, such as the messages size and use Web Services Dynamic Discovery (WS-Discovery) for locating services on a local network instead of centralized service directory. These restrictions ensure that web services run properly on devices with limited resources. DWPS is focused on the development of applications in devices with the IP protocol and allows basic functions with web services, such as the use of reliable mechanisms for sending and receiving messages, the dynamic discovering of web services with WS-Discovery [46], the specification of a web service and the subscription/reception of events from another web service. There are other research works that have used DPWS to develop services platforms in ubiquitous scenarios with good results, which reinforces the selection done [47].

**Figure 2.**
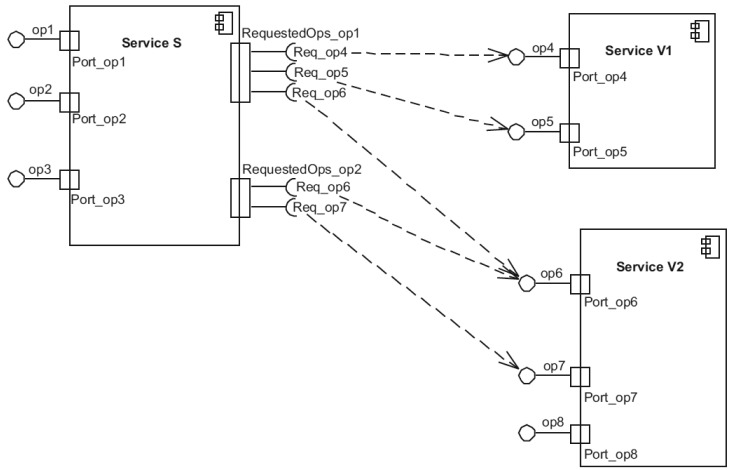
A UML component diagram for modeling a system

An IoT service can be represented using a UML (Unified Modeling Language) component diagram, as we can see in Figure 2. The component diagram allows us to achieve a global vision of how the set of services can be combined to implement a composite application. Each service is a black box that exposes the set of operations that can be invoked by a consumer service without revealing its implementation. Thus, consumer services do not know how that service carries out its operations, even if these operations are simple or composite.

Each IoT service requires the implementation of the set of operations that accomplishes the functionality of the service and the specification of the service definition, which determines a description of its functionality, its collaborative behavior, its configuration properties and other service attributes. There are three main elements associated with the service definition of each IoT service that are conforming to the formal definition introduced in previous Section 3.1 (Definition 1). These elements are service contract, service composition map and service configuration.

Each IoT service according to DOHA has a public service contract. The service contract describes the purpose of the service defined in *Ps(S_i_)*. The set of operations of the service is equivalent with the provided interface of the service definition, *Ip(S_i_)*. This description is public and accessible to service consumers when they want to use the service.

The service composition map defines the composite operations of services. As described above, each composite operation has its own composition map. Then, the service composition map of a given service will be the set of composition maps of its operations. The set of requested operations in the service composition map is equivalent with the *Ir(S_i_)* of the service definition. This document is private, and it is accessible only by its own service.

Finally, the service configuration specifies the configuration parameters required to execute the service properly, as well as the execution attributes of the service; e.g., the physical location of the node where the service is running, the type of connectivity, the service life time or QoS properties, among others. However, it also includes an identification of the service instance that can be queried by service consumers and other additional information about its service content. Then, these values can be different among different instances of the same service, *i.e.*, different instances of the same service will have different service configuration. The identification and configuration properties of each service included in this set are equivalent with the elements *Id(S_i_)* and the service attributes *At(S_i_)* specified in the service definition. In some case, the service configuration can contain non-typed information in the form of file resources, such as a pdf document or image that can be downloaded through a URL.

Using Definition 1 and the concepts of contract, map and configuration, we can define each IoT service in a formal way. To do this, we have used a tuple set, encapsulating service contract, composition map and service configuration info, as we can see in Definition 6.

(6) $$\begin{array}{c} {\text{IoT service}}_{i}=<\;Id_{i},Ps_{i},Ip_{i},Ir_{i},At_{i}>\\ \text{edge}({\text{o}}_{{\text{op}}_{\text{v}1}},{\text{op}}_{\text{v}2},\text{v}2)\to {\text{complexDeg}(\text{op}}_{\text{v}1})>\text{complexDeg}({\text{op}}_{\text{v}2}) \end{array}$$

All the specification documents of the service, *i.e.*, contract, composition map and configuration are stored in XML (Extensible Markup Language) files according to an XSD (XML Schema Definition) schema. The external consumer services will process and interpret the service data by first acquiring the metadata from XSD. In particular, the service contract is enclosed in a WSDL document for the DPWS implementation of DOHA, as is usual in web service technologies.

In the case of device services, a description of hardware resources and service execution can be important to consumer services, especially when a fusion algorithm is performed with particular time restrictions (even real time). Service consumers can obtain the required typed information from the specification documents of the service, especially from the service configuration. In addition, a standardize model to describe the hardware resource can be included in the service configuration to further simplify their reading and improve their interoperability. Hu *et al.* [41] discusses some of the possible standards for specifying information about sensors. Thus, the IEEE 1451 family offers a standard definition for describing hardware resources, the Transducer Electronic Data Sheet (TEDS), which is essentially an electronic replacement of a transducer data sheet on paper. Device services can store a TEDS when it is available to provide more information about the sensor or actuator enclosed in the service that can be processed directly by service consumers (e.g., a service fuser). In other cases, a data sheet of the manufacturer device in a pdf document is downloaded from a URL.

## 4. Case Study

Summing up the concepts previously presented in the paper, the proposed data fusion acquisition method is based on a static composition model with dynamic selection of service implementations or instances. The composition model determines several parameters to the correct execution of composite operations. The composition map establishes how the composition is carried out and helps to avoid cycles, maintaining the correctness of the degree of complexity associated with the operations and services involved in the collaboration.

To illustrate the proposed data fusion model based on service composition, a weather forecast system was developed. This system is able to determine a local weather prediction from the measurement of atmospheric conditions of a specific location. To achieve this, several device services were implemented to know the atmospheric conditions of temperature, pressure and humidity. These device services were replicated for two reasons: (1) to ensure the correctness of the measurements; and (2) to detect sensors/actuators that are not working properly. Once the measurements were verified, an algorithm was applied to estimate a local weather prediction. The evaluation of the correctness and the determination of weather were carried out by the implementation of other IoT services applying data fusion schemes, as we detail below.

### 4.1. Hardware Devices

Tests have been executed in six different distributed embedded devices, five Raspberry-Pi's and one Android tablet. Each Raspberry-Pi will be called R1, R2, R3, R4 and R5 in the rest of the paper. The Raspberry-Pi is a powerful embedded device with a processor ARM1176JZF-S 700 MHz, 512 Mb RAM, Ethernet 100 and Java JDK 1.7 running over Linux Debian [48]. The Android tablet, henceforth called AT, has been used to interact with the users. It is a T30s with four cores, 1 GB RAM and Wi-Fi connection running over Android 4.1.2.

**Figure 3.**
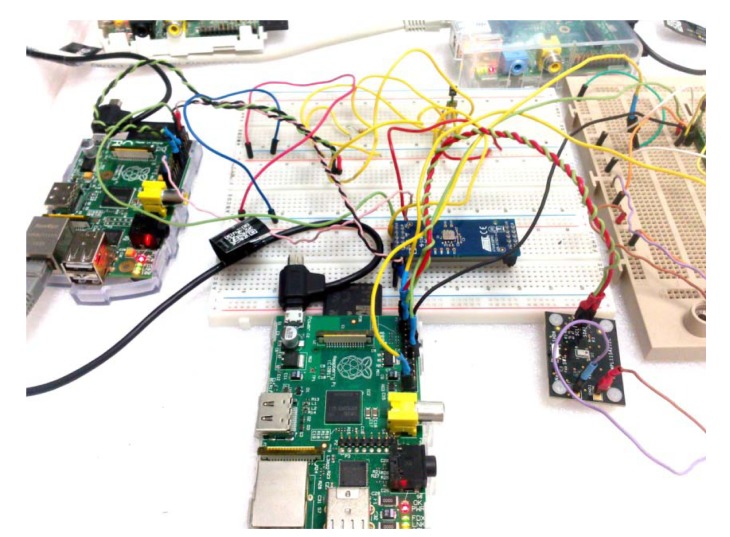
Devices used in the prototype development.

Three different types of sensors have been connected to Raspberry boards to measure the values of humidity, temperature and pressure. The sensors used are FreeScale MPL115A2 Digital Barometer (FreeScale) [49], Atmel AVR4210 Digital Pressure Sensor (Atmel) [50] and the Sensirion SHT7x Humidity and Temperature Sensor Integrated Circuit (Sensirion) [51]. We have used three Raspberries to connect these sensors, R1, R2 and R3. An Atmel AVR4201 and a Sensirion SHT71 are connected to R1 and R2, whereas a FreeScale MLP115A2 and a Sensirion SHT75 are connected to R3. Figure 3 shows the Raspberries R1 and R3 and some of the sensors connected to them.

Tables 1 and 2 show the properties of the sensors connected to R1, R2 and R3, as well their relation with the type of value measured, *i.e.*, humidity, pressure or temperature. The values of humidity were measured using the Sensirion sensor. The values of pressure were measured using the Atmel sensor in R1 and R2 and the FreeScale in R3. Finally, the values of temperature were obtained as the mean between the value of temperature read in Atmel and Sensirion in R1 and R2 and FreeScale and Sensirion in R3. The objective of the paper is not to analyze the quality of each of these sensors, although we recommend that the reader revise the corresponding datasheet to obtain extended information.

### 4.2. IoT Services

A set of scalable services must be designed and prepared to build the weather forecast system. Firstly, a device service was implemented for each sensor, obtaining three device services, temperature device service (TDS), humidity device service (HDS) and pressure device service (PDS). In addition, these services are replicated three times, because the use of multiple sensors may increase the accuracy with which a quantity can be observed and characterized [11]. Then, we had different instances of each device service, *i.e.*, three different objects with the same functionality: TDS1, TDS2 and TDS3, to measure the temperature; HDS1, HDS2 and HDS3 to measure the humidity; and PDS1, PDS2 and PDS3 to measure the pressure.

The identification of services is one of the most challenging areas of research in IoT. In the case of DOHA, we resolved it in the previous stage of services definition using the formal schema to represent the ubiquitous services presented in Section 3.1.

We fused the information obtained by replicated services of each kind of sensor using an N-Version algorithm to ensure the correctness of the values in new services, *i.e.*, the device services requested are replicated N times. Fusing multisensor data provides us significant advantages over single-source data [52]. The services which apply the N-Version algorithm are temperature N-Version service (TNVS), humidity N-Version service (HNVS) and pressure N-Version service (PNVS), all with *N* equals three. Algorithm 1 shows a pseudocode for this kind of service. The operation *requestSensorValue()* corresponds with the specific operation in the device service, which returns the value of the sensor: the operation *getTemp()* of the service TNVS invokes *getTValue()* in each TDS; the operation *getHumidity()* of the service HNVS invokes *getHValue()* in each HDS; and the operation *getPressure()* of the service PNVS invokes *getPValue()* in each PDS.


**Algorithm 1.** N-Version services: operations *getTemp(), getHumidity()* and *getPressure()*.
Input: threshold (double)Output: value (double)/* Stage 1: request sensor values */for s:Sensors do value[s] = requestSensorValue();/* Stage 2: validate sensor values using threshold */for n = 1 to numSensors-1 do for i = n + 1 to numSensors do  if abs((value[n]-value[i]) > threshold)   version[n] = 0/* Stage 3: calculate nVersion value */for n=1 to numSensors if (version[n]! = 0)  sumVersion + = version [n]  nVersion++return nVersion > 0 ? sumVersion/nVersion : 0


The weather forecast service (WFS) uses N-Version services to apply an algorithm based on the probability of determining the weather that users could find outside their house or when they leave home. The algorithm implemented in the WFS is an extension of the Zambretti forecaster [53], which uses measurements of pressure and wind direction to predict the weather. We have modified the original Zambretti algorithm, and our WFS also uses temperature and humidity to improve the weather prediction. Algorithm 2 shows the pseudocode of the operation *getPrediction()* of this service.


**Algorithm 2.** Weather forecast service: operation *getPrediction()*.
Input: temperature (double), pressure (double), humidity (double)Output: weather-forecaster-index (int)/* Calculate the Cloud Base value */dewPoint = calculateDewPoint(temperature, humidity);cloudBase = calculateCloudBase(temperature, dewPoint);/* Calculate the prediction */return matchPreddiction(pressure, cloudBase);


The values of temperature, pressure and humidity that the Algorithm 2 receives as inputs correspond with the results obtained after the execution of each N-Version services algorithm. The value of temperature is the result of execute the operation *getTemp()* in the TNVS; humidity is the result of execute the operation *getHumidity()* in the HNVS; and pressure is the result of execute the operation *getPressure()* in the service PNVS.

Finally, the climate service (CS) uses the information provided by WFS and N-Version services to generate a complete pack of weather information to give better and more comprehensive suggestions to the users. The operation that this service implement has been called *getClimate()*.

**Figure 4.**
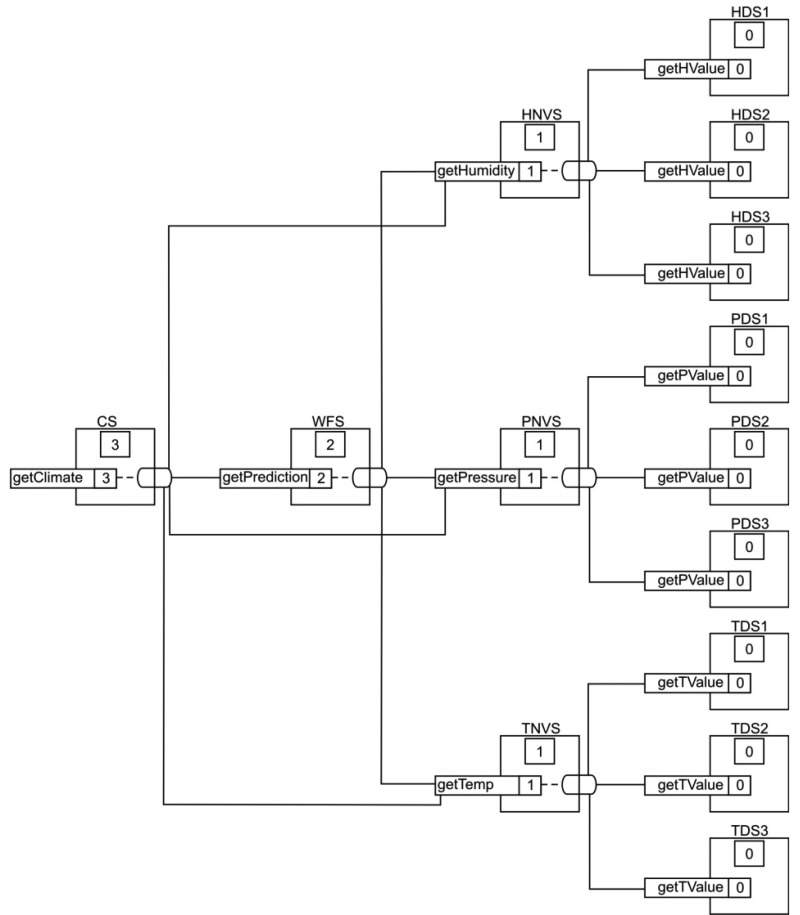
Full composition map of the system.

Services collaborate with each other with the common goal of fusing sensor data and giving complete information to the users. Each service with composite operations will have its own composition map with a specific value of degree of complexity, which depends on the maximum degree of all of its composite operations. Figure 4 shows the full composition map of the system and the relation between the degree of complexity of each service, its operations and its position in the graph of the full system composition map. As we can see in this figure, N-Version services, HNVS, PNVS, TNVS, WFS and CS are composite services. Moreover, their composite operations are fusing information that is obtained from other services with a lower degree of complexity. Then, in this example, all of these composite services are working as fuser services.

The use of composite operations in fuser services for data acquisition favors the scalability of the system, *i.e.*, it does not matter if fuser services change or if new services have been included: they will be able to get the data following the service composition model. The system does not need centralized servers, and it provides an easier mechanism to add new services and devices, increasing the set of data sources. Thereby, the developer will have more information available to implement their algorithms.

The data fusion process is part of the collaborative model of the service platform, and therefore, it is executed in a distributed, lightweight manner over the services network. Moreover, data can be obtained from services as data sources, including time constraints. This allows us to consider the setting of time parameters as QoS properties for the development of data fusion algorithms in the context of soft real-time. Then, in this case, a fuser service could ensure their clients timely fused information, providing additionally a maximum delay time to achieve a response and the minimum sampling time required for invoking its operations.

**Figure 5.**
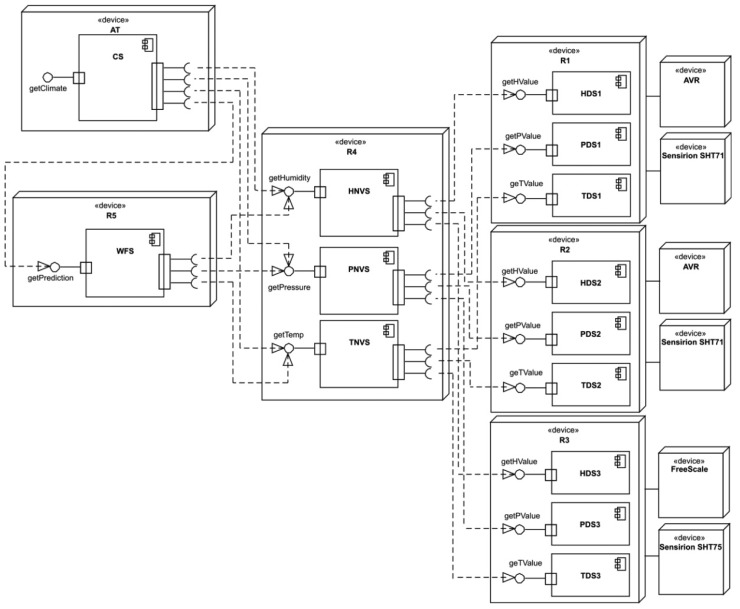
Deployment diagram of the system.

All services introduced in this section have been deployed using devices enumerated in Section 4.1. Services have been implemented using DOHA and are interconnected with a local area network. TDS1, HDS1 and PDS1 were running in R1; TDS2, HDS2 and PDS2 in R2; TDS3, HDS3 and PDS3 in R3; TNVS, HNVS and PNVS in R4; WFS in R5 and CS in AT. Figure 5 shows the deployment diagram of the system.

### 4.3. Evaluation

To evaluate the proposed approach, we have measured the execution time of all operations involved in the services of the case study. These values help us to analyze the data acquisition mechanism implemented by the service composition model. Controlling the maximum response time, we can also guarantee the soft real-time execution of the operations.

The analysis of the case study has been carried out taking into account two alternative execution modes of the services:

- Request/response mode (RRM): In this mode, when an operation of a service is invoked by a consumer service, the service executes its implementation, including the invocation of all required operations, composite or simple, in other services, according to its composition map. If the required operations are also composite, then the corresponding nested operations are also called sequentially. The flow control of invoker is then blocked, until the operation of the service has finished and a response is returned.
- Virtualized mode (VM): In this mode, the service virtualizes the resources, status or data belonging to other required services by saving a temporary copy of them in its memory. Then, after an operation is requested by a consumer service, the service executes its implementation, substituting the invocation of required operations defined in its composition map by a call to local operations, which provide the corresponding previous saved values in memory. Consequently, the service may respond by almost immediately returning the expected results, reducing also significantly the execution time of the operation. To achieve that, a background process in the service has to be executed periodically in order to invoke all of the required operations defined in the composition map to update the memory with the most recent values.

Two different types of tests have been executed: (1) a network traffic analysis during the execution of the services involved in the case study; and (2) measuring the execution time of each operation for each service contained in the case study.

#### 4.3.1. Network Traffic Analysis

**Figure 6.**
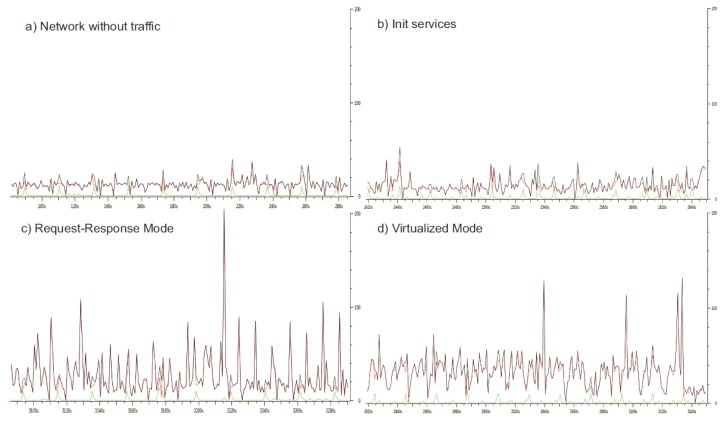
Network traffic evolution in four states: (**a**) without traffic; (**b**) with the initialization (init) of services; (**c**) in request-response mode and (**d**) virtualized mode.

An analysis of the network traffic is performed when the network is resting, during the initialization of services and during the execution of services in the two possible modes, RRM and VM. Figure 6 shows the differences between these four states.

The graphic in Figure 6a shows the normal state of the network in a resting situation, *i.e.*, without any service in execution. However, Figure 6b shows an increase of the traffic along the initialization of services. This increase in the traffic network is directly related to the use of the network done by IoT services. Since IoT services use DPWS as underlying communication middleware, during their initialization, they send hello messages by broadcast using UDP, which is softly higher than in Figure 6a.

With services running in RRM mode, Figure 6c shows higher network traffic with the presence of several peaks. These peaks represent the execution of composite operations with a high degree of complexity. This happens because composite operations with a high degree of complexity trigger the execution of other requested operations in a chain with the corresponding increasing in the number of messages in the network.

In the case of VM execution mode in Figure 6d, the network traffic is increased with respect to resting mode, but without the presence of peaks. The reason is because all composite operations are periodically executing their requested operations in a background process to save in memory the most recent results regardless of whether such composite operations are invoked. This enables a faster response to the invoker services and provides a load balancing for the network.

#### 4.3.2. Service Execution Analysis

An analysis of the service execution is carried out by evaluating the execution time of the operations of each service involved in the case study considering the two proposed execution modes, request-response (RRM) and virtualized (VM). The experiment consists of starting the services and then tracks the executions of their operations repeatedly in order to measure their execution times.

Two types of results are presented. On the one hand, Table 3 includes a summary of the performed experiments, providing the results of the average time execution, the standard deviation and WCET obtained in both modes for each operation of the services used in the case study. On the other hand, a graphic is presented to show the variances of the execution time for each analyzed operation.

Some important decisions have to be considered in the elaboration of Table 3. The first values of execution time in VM mode are outliers and have been deleted in order to not impact the statistic.

**Figure 7.**
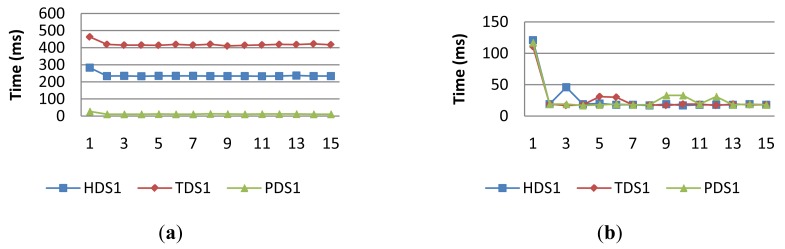
Execution times of device services located in R1 in (**a**) RRM mode and (**b**) VM mode.

Figure 7 shows the execution time of simple operations for PDS, TDS and HDS device services in order to obtain the corresponding sensor values of atmospheric pressure, temperature and relative humidity in the Raspberry-Pi R1. These device services shared the same I2C bus to access the BMP085 and SHT71 chips that had the physical sensors. Then, the readings of the sensors had to be performed sequentially.

The experiments were carried out in two modes, RRM and VM. In this case, RRM indicates that the simple operation of a device service enables a request-response message to the physical sensor over the I2C bus. Likewise, VM mode implies that the device service has the virtual value of the physical sensor in memory. Then, the invocation of each simple operation returns immediately with the temporarily saved value of the sensor, which is updated in the device service periodically by a background process.

Figure 7a and Table 3 show that the execution time of device service operations located in R1 in RRM is not high, less than 500 ms for each simple operation. However, we have found differences in the execution time of the simple operation among services for three reasons. Firstly, there was a bottleneck in the access of the I2C bus for the sensor reading, which depends on the order of requests. Secondly, with a lower influence, the time involved in the ADC conversion depends on the resolution of ADC and the sampling period of the sensor. This explains the delay of the operation execution for TDS and HDS with respect to PDS. However, in addition, TDS has a higher execution time than PDS, because it fuses the temperature reading of both chips. In fact, the temperature value is obtained as the medium values of two sensors, the Atmel in R1 and R2 or the Freescale in R3 and the Sensirion located in each Raspberry-Pi. Figure 7b shows the execution time of device service operations in VM mode. In this case, we observed a decrease in execution time with the same scale, since the operation of device services returns immediately.

**Figure 8.**
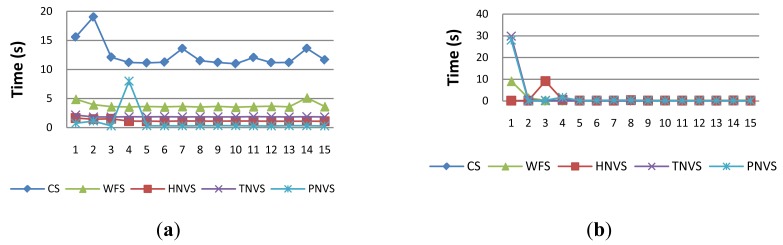
Execution times of the composite services in (**a**) RRM and (**b**) VM.

Figure 8 shows a comparison between the execution of the operations specified in Table 3 for CS, WFS, HNVS, TNVS and PNVS in both modes, RRM and VM. In the case of N-Version services with a degree of complexity of one, HNVS, TNVS and PNVS, each operation “*get*” requires the invocation of a simple operation in three different instances of the same device service to fuse data, according to Figures 4 and 5; e.g., the operation *getPressure()* in PVNS must invoke the operation *getPValue()* in three different PDS. In RRM mode, the execution time of an operation in an N-Version service is higher, because it has to invoke and execute sequentially all of the required operations on device services. Likewise, this behavior is also found for the execution of *getPrediction()* of WFS with nine operations and *getClimate()* of CS with 19 operations. The fluctuations in execution time of some iterations observed in Figure 8a are caused mainly by noise in the network.

In contrast, in VM mode, the execution time of an N-Version service is lower, since the execution of required operations is solved only by local operations in the scope of the same service. The minor fluctuations of the execution time observed in Figure 8b, especially at first executions, respond to the interference that the background process, responsible for updating the memory with the most recent values, can cause to the foreground process. This behavior is also found for the operation *getPrediction()* of WFS and *getClimate()* of CS.

We can conclude that the execution time of an operation in RRM mode depends directly on the degree of complexity of that operation. A higher degree of complexity imposes higher execution time, because the number of requested operations grows, as well. In fact, the execution time of an operation is influenced by the number of operations invoked during the execution of an operation, the network delay round-trip or the end-to-end time required for the invocation of each requested operation and, finally, the execution time of each requested operation. However, in VM mode, the execution time of an operation is much lower, depending only on the implementation of that operation, because its execution is local.

The execution time observed for an operation of a particular service determines also the rate at which that operation can be invoked. This time defines the sampling period of an operation, a parameter that can be important to setup a data fusion algorithm, for example, to fix the time for sustaining data provision from a data source. A good first choice is to set the minimum sampling period for each operation equals to its WCET, because WCET guarantees an upper bound for every possible execution of the operation. Then, the operation cannot exceed ever this deadline, and regular behavior can be achieved for the specific data fusion algorithm.

In Table 3, we have seen that in RRM mode, the operations of device services have a WCET with a magnitude order of hundreds of milliseconds; the N-Version services have a WCET with an order of one second; the WFS service has an order of five seconds; and finally, a CS service has an order of ten of seconds. However, the obtained WCET was measured experimentally from the execution times data set of every operation recorded during the test executions, and some outliers of the data set can have a negative impact, over-estimating the WCET determination. The result can be a very pessimistic value, far away from the average execution time, which leads to a significant waste of computing resources; e.g., the WCET for *getPressure()* of PNVS is greater than the WCET for *getPrediction()* of WFS. A lower upper bound could be considered to optimize the WCET closer to the average execution time, which we call the best worst-case execution time (BWCET). The BWCET is determined by evaluating the maximum of the 99% confidence interval for a standard normal distribution of execution times, which give us a 99% probability that the execution time of an operation has a value within the interval. Table 3 shows the BWCET for each operation of the study case, with a value in all cases closer to the average execution time, allowing a better utilization of computing resources.

The WCET and, better, the BWCET can be used to set also the sampling period of each operation of a specific service in VM mode, in spite of the execution time being much lower. This is because the service is updated by the background process with a period of execution, which must be equal to the sampling period of the operation. This period is larger than the execution time of the operation and depends on the number of requested operations in other services and their sampling periods. In VM mode, there is a limited interchange of messages between services compared with RRM mode, because each service is responsible for performing the requests of the required operations in provider services autonomously and at regular rate, ensuring even the satisfaction of soft real-time constraints. This contributes to improving the network load, as we have observed in Figure 6d.

In summary, from the analysis of the results obtained in this case study, we can set some guidelines and recommendations for the implementation of data fusion programs according to the proposed distributed service-based approach:

(1) The building of a scalable system for data fusion processes is safer when a service composition model is considered.
  A service composition model, such as is presented in this paper, gives a correct way to build scalable services. In general, the service composition model gives mechanisms to improve the interconnections and the communications between services, addressed by the services in the case of orchestration or evaluating the possible interconnections in each service in the case of choreography. In our case, the proposed service composition model goes beyond providing a way to verify globally all of the services of the system. This is particularly interesting in the implementation of data fusion processes, especially when the system must be built gradually at different times without the need of re-implementing the system whenever a new data source is included or a new fusion algorithm is implemented.
(2) It is preferable to build systems that contain services with a low degree of complexity of operations.
  When the degree of complexity of an operation is low, the operation does not have to invoke many operations in the chain. Hence, its execution time may have a lower magnitude order, as well as its sampling period. However, the system is completely scalable, and it is possible to add services with a higher degree of complexity of operations assuming the costs in execution time.
(3) When the service contains operations with a high degree of complexity, the execution in VM is preferred rather than the execution in RRM.
  When the degree of complexity of an operation in a service is high, the invocation of this operation in RRM is penalized for two reasons. First, the invocation of this operation can require enough network resources (e.g., high bandwidth or low network noise), because the number of messages transmitted to the network can be very high in short time intervals, provoking peaks of network load, that it may not be assumed. Second, the flow control of the invoker consequently blocks large amounts of time, because it is suspended until the response is received. An excessive blocking time of flow control favors the building of poor and underutilized systems.
  The use of VM for the execution of a service can benefit the overall execution of the systems, because the network load is shared among all services instead of addressed by a specific service. Each service is responsible for keeping updated the required data acquired from other services according to its composition map. In addition, the blocking of the flow control of any invoker operation to the service is reduced significantly, which consequently improves the responsiveness of the system.
(4) In a real-time context it is preferable to fix the sampling period of every operation of each service, especially composite operations, with WCET or, better, BWCET, depending on the stringent conditions of real time.
  The idempotence property of the service ensures that the service can manage multiple requests at the same time without changing or otherwise affecting the data. However, a continuous invocation of an operation with a rate greater than the minimum sampling period may overload the service without gaining useful information, since the service does not have sufficient time to achieve or update the results. The application of real-time constraints into a data fusion system can benefit the global execution of the system, performing better control of computing resources of the system. Depending on the criticality conditions of the system, the engineer can set the sampling period with WCET when the managed data or involved services are critical or with BWCET in another cases.

## 5. Conclusions

The relevance of the IoT in the scope of pervasive computing is growing. The number of embedded devices present currently in daily life is increasing in an exponential way. At home, at work and even in our relationship with other people, we use continuously multiple interconnected embedded devices. The information provided by these kinds of objects and devices must be interpreted and, sometimes, merged to obtain richer information.

A lot of research works pose data fusion process using complex algorithms, always centralized on a powerful computing node, where data is acquired and processed from heterogeneous sources. In this paper, we have proposed a novel method to implement a distributed data fusion acquisition using a lightweight service composition model, which ensures the correctness of collaborations without a cyclic behavior. This method allows working with data in a distributed, decentralized manner. The high level model of representation proposed abstracts the typical underlying complexity in IoT scenarios due to its heterogeneity of devices. Thanks to this high level of abstraction, developers can use the concept of service and the interaction among services to design the IoT scenario.

The interaction among services is carried out using a well-defined service composition model. This allows one to use basic elements or high level software units during the design of an IoT system. At the service level, each service is responsible for acquiring data from external services by means of its composite operations in order to manage, combine, fuse or build new information, which, in turn, is shared with any other service that claims it. Using the service composition model adds relevant QoS properties to the system, such as a bounded execution time and sampling period. If developers know what the WCET or BWCET of a composite operation is, they can add soft real-time restrictions to the system execution.

The distributed character of the proposed approach for data fusion makes the service model very scalable, a really important aspect in the development of current and future applications, which will be deployed in embedded devices. Moreover, the proposed data fusion model allows developers to have a macroscopic view of the complete system or almost a partial view of the involved services in the system. In software environments where there are many embedded devices with several restrictions, such as low resources, memory or process power, and communications deadlines, this panoramic vision helps developers to optimize the use of the whole system. Furthermore, developers can test their services before deploying them in a real system, analyzing their network use, their composite behavior, the maximum execution time and other aspects related to the collaboration between services relevant for the real execution of the full system.

We are working to improve the service composition model presented in an IoT environment using semantic information, QoS properties and ontologies to facilitate the dynamic selection of services at runtime. This will transform the data fusion acquisition model presented in this paper into a richer dynamic tool to use in the IoT scope, among others.

## Figures and Tables

**Table 1.** Properties of the sensors from the manufacturer datasheet connected to the Raspberry-Pi 1 and Raspberry-Pi 2.

	**Type**	**Sensor Chip**	**Resolution**	**Accuracy**	**Repeatability**	**Output**
Humidity	Sensirion	SHT71	12 bits	±3.0	±0.1	2-wire interface
Pressure	Atmel	BMP085	0.01	±0.2	-	I2C
Temperature	AVR/Sensirion	BMP085/SHT71	0.1/14 bits	±0.5	±0.1	I2C/2-wire

**Table 2.** Properties of sensors from manufacturer datasheet connected to the Raspberry-Pi 3.

	**Type**	**Sensor Chip**	**Resolution**	**Accuracy**	**Repeatability**	**Output**
Humidity	Sensirion	SHT75	14bits	±1.8	±0.1	2-wire interface
Pressure	FreeScale	MPL115A2	0.15	±1 kPa	-	I2C
Temperature	FreeScale/Sensirion	MPL115A2/SHT75	0.1/14bits	±0.1	±0.1	I2C/2-wire

**Table 3.** The table shows the average execution times, standard deviation and worst-case execution time (WCET) for each operation of the services of Weather Forecast System. For request/response mode (RRM) mode, the table includes the value of BWCET, which is explained in the text. VM, virtualized mode; BWCET, best worst-case execution time; PDS, pressure device service; TDS, temperature device service; HDS, humidity device service; PNVS, pressure N-Version service; TNVS, temperature N-Version service; HNVS, humidity N-Version service; WFS, weather forecast service; CS, climate service.

**IoT Service**	**Operation**	**RRM mode**				**VM mode**		
	
		**Average Execution Time (s)**	**Standard Deviation (s)**	**WCET (s)**	**BWCET (s)**	**Average Execution Time (s)**	**Standard Deviation (s)**	**WCET (s)**
PDS1	getPValue	0.013	0.004	0.027	0.015	0.021	0.006	0.117
TDS1	getTValue	0.420	0.012	0.463	0.428	0.026	0.024	0.111
HDS1	getHValue	0.238	0.013	0.283	0.246	0.027	0.027	0.121
PNVS	getPressure	0.910	1.975	8.001	2.224	0.313	0.409	1.687
TNVS	getTemp	1.873	0.084	2.169	1.929	0.207	0.095	0.533
HNVS	getHumidity	1.196	0.181	1.640	1.317	0.810	2.309	9.148
WFS	getPrediction	3.812	0.500	5.135	4.144	0.390	0.278	1.353
CS	getClimate	12.486	2.223	19.026	13.964	0.001	0.001	0.004
